# Cell Division in Apicomplexan Parasites Is Organized by a Homolog of the Striated Rootlet Fiber of Algal Flagella

**DOI:** 10.1371/journal.pbio.1001444

**Published:** 2012-12-11

**Authors:** Maria E. Francia, Carly N. Jordan, Jay D. Patel, Lilach Sheiner, Jessica L. Demerly, Justin D. Fellows, Jessica Cruz de Leon, Naomi S. Morrissette, Jean-François Dubremetz, Boris Striepen

**Affiliations:** 1 Department of Cellular Biology, University of Georgia, Athens, Georgia, United States of America; 2 Center for Tropical and Emerging Global Diseases, University of Georgia, Athens, Georgia, United States of America; 3 Department of Molecular Biology and Biochemistry, University of California-Irvine, Irvine, California, United States of America; 4 UMR 5235 CNRS, Université de Montpellier 2, Montpellier, France; University of Vermont, United States of America

## Abstract

Apicomplexa are intracellular parasites that cause important human diseases including malaria and toxoplasmosis. During host cell infection new parasites are formed through a budding process that parcels out nuclei and organelles into multiple daughters. Budding is remarkably flexible in output and can produce two to thousands of progeny cells. How genomes and daughters are counted and coordinated is unknown. Apicomplexa evolved from single celled flagellated algae, but with the exception of the gametes, lack flagella. Here we demonstrate that a structure that in the algal ancestor served as the rootlet of the flagellar basal bodies is required for parasite cell division. Parasite striated fiber assemblins (SFA) polymerize into a dynamic fiber that emerges from the centrosomes immediately after their duplication. The fiber grows in a polarized fashion and daughter cells form at its distal tip. As the daughter cell is further elaborated it remains physically tethered at its apical end, the conoid and polar ring. Genetic experiments in *Toxoplasma gondii* demonstrate two essential components of the fiber, TgSFA2 and 3. In the absence of either of these proteins cytokinesis is blocked at its earliest point, the initiation of the daughter microtubule organizing center (MTOC). Mitosis remains unimpeded and mutant cells accumulate numerous nuclei but fail to form daughter cells. The SFA fiber provides a robust spatial and temporal organizer of parasite cell division, a process that appears hard-wired to the centrosome by multiple tethers. Our findings have broader evolutionary implications. We propose that Apicomplexa abandoned flagella for most stages yet retained the organizing principle of the flagellar MTOC. Instead of ensuring appropriate numbers of flagella, the system now positions the apical invasion complexes. This suggests that elements of the invasion apparatus may be derived from flagella or flagellum associated structures.

## Abstract 2

Apicomplexan parasites undergo cell division using an evolutionarily conserved mechanism first described in the positioning and assembly of flagella in algae.

## Author Summary

Malaria, toxoplasmosis, and related diseases are caused by infection with unicellular parasites called Apicomplexa. Their name refers to the elaborate invasion machinery that occupies the apical end of the parasite cell. This apparatus allows the parasite to force its way into the cells of its host, and to deliver factors that will manipulate host cell structure, gene expression, and metabolism. Once in the host cell the parasite will begin to grow. The parasite replicates its genome and organelles numerous times and then loads these various elements into numerous daughter cells that will further spread the infection. Here we report a fiber that coordinates the daughter cell budding process. The fiber links the centrosome, which controls the mitotic spindle, and the genome with the microtubule organizing center of the budding daughter. Parasite mutants lacking the proteins that build the fiber fail to form daughter cells at the earliest step. The fiber and its components are remarkably similar to fibers that coordinate flagella in algae. While Apicomplexa are not flagellated (with the exception of certain gamete stages) they evolved from flagellated algae. We propose that elements of the invasion apparatus evolved from the flagellum or flagellum associated structures.

## Introduction

Apicomplexa are protozoan parasites responsible for numerous human and veterinary diseases. Human pathogens in this phylum include *Plasmodium*, the causative agent of malaria, *Toxoplasma*, an opportunistic pathogen that causes encephalitis in immunocompromised individuals and congenital toxoplasmosis, and *Cryptosporidium* one of the most important causes of severe early childhood diarrhea around the world. Apicomplexa are obligate intracellular parasites that follow a stereotypical propagation cycle. A motile zoite stage seeks out and invades a suitable host cell and in this process establishes a novel compartment, the parasitophorous vacuole, that houses the parasite during its intracellular development [1]. Parasites replicate and ultimately produce a new generation of zoites that destroys the host cell upon egress and fan out to infect new cells. Apicomplexans have adapted to tissue and host cell niches as varied as red blood cells, intestinal epithelial cells, macrophages and lymphocytes, or neurons.

**Figure 1:**
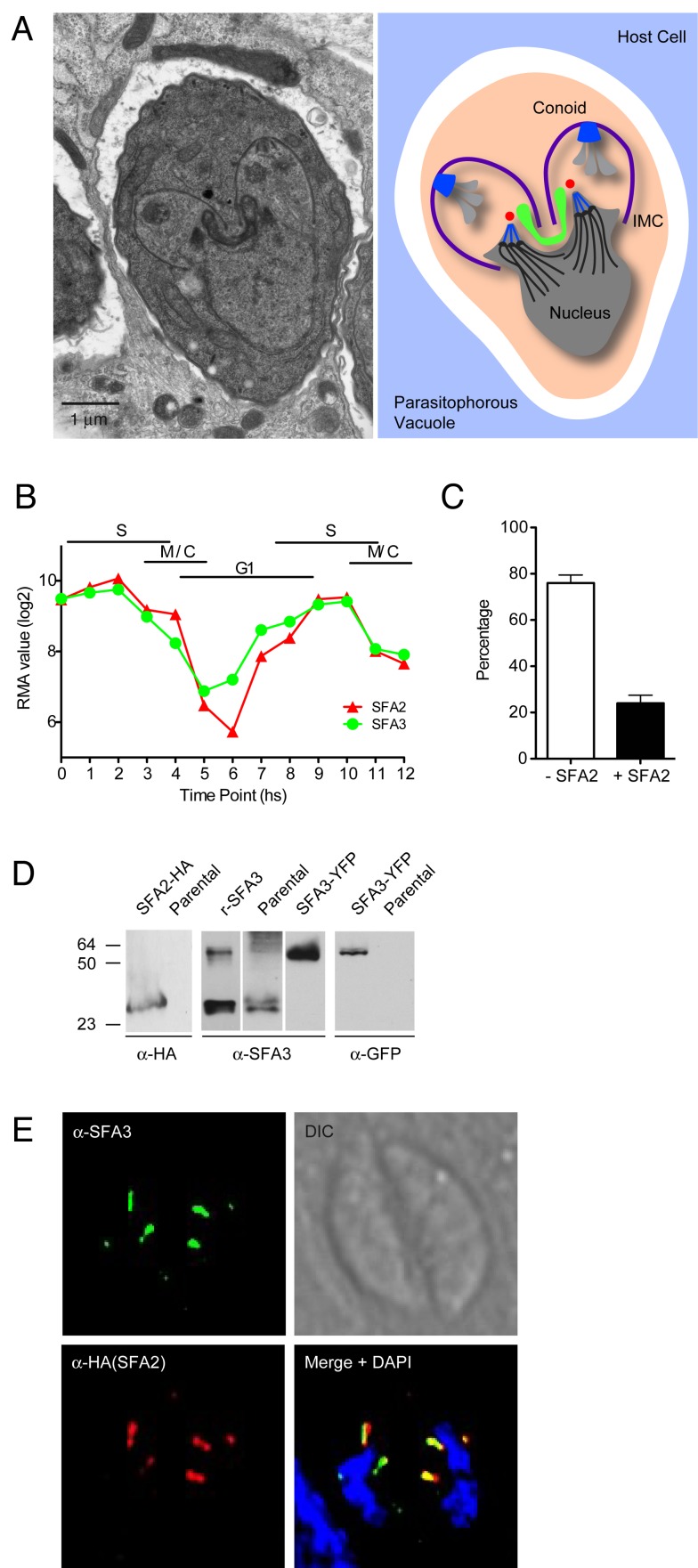
*T. gondii* tachyzoites express two SFAs that localize to a structure close to the nucleus. (A) Electron micrograph and schematic of a dividing *T. gondii* parasite. Two daughters are assembled within a mother cell. Centrosomes are shown in blue, apicoplast in green. (B) Robust multi-array averages of SFA2 and SFA3 transcripts over two consecutive division cycles based on dataset collected by Behnke and colleagues [19],[20]. (C) Parasites do not express TgSFA2 at all times. Parasites expressing SFA2-HA were scored by IFA using anti-HA antibody in an asynchronous parasite population (*n* = 250). (D) Western blot analysis shows a 30-kDa band when probed with anti-HA antibody in SFA2-HA transgenics but not the parental strain. Anti-SFA3 antibodies recognize the recombinant protein used for immunization (rSFA3), as well as the native protein in parental parasites. Lower mobility bands of approximately 60 kDa likely correspond to dimers formed both by the recombinant and native SFA3 proteins. Anti-SFA3 antibodies also recognize YFP tagged SFA3 in the endogenously tagged TgSFA3-YFP strain. The expected masses for SFA3 and SFA3-YFP are 36 kDa and 56 kDa, respectively. Anti-GFP antibodies specifically recognize SFA3-YFP in the endogenously tagged strain, but not in the parental strain. (E) IFA of SFA2-HA parasites stained with anti-SFA3 and anti-HA antibody and DAPI. Note that both SFA proteins localize to two fiber-like structures per parasite.

The budding mechanism used by apicomplexans appears to be the key to their ability to scale their reproductive output to the size and biology of the specific host cell [2]. In this process, many species including the malaria parasite, deviate from the conventional cell cycle and pass through DNA synthesis and nuclear mitosis numerous times amassing a large number of genomes. Coinciding with the last round of mitosis, daughter buds are assembled and each nucleus is packaged into a new zoite. It is not understood how the parasites match the number of nuclear genomes with emergent daughter buds and how buds are placed and assembled correctly. The bud is scaffolded by microtubules that emanate from a newly formed apical microtubule organizing center (MTOC) (shown in blue in Figure 1A for *T. gondii*) [3]. These microtubules anchor the inner membrane complex (IMC, purple), an assemblage of membranous and cytoskeletal elements that establishes cell shape and is critical to the parasite's gliding motility [4]. The MTOC is thought to be a ring structure and can be further elaborated by additional elements including the conoid [3],[5],[6]. The MTOC also organizes the specialized apical secretory machinery that delivers proteins for host cell invasion and modification. Secretion of these organelles occurs at the extreme apex and through the ring [7],[8].

The centrosome has been demonstrated to organize parasite chromosomes and some organelles. Interestingly, both in *T. gondii* and *P. falciparum* chromosomal centromeres are constantly tethered to the centrosome [9],[10]. A similar physical association with the centrosome has been described for the apicoplast and the Golgi [11]–[13]. We hypothesized that a second tether linking the centrosome to the daughter bud MTOC and the associated invasion machine could provide a robust mechanism for cell assembly. In this study we use *T. gondii*, which divides using the simplest internal budding process in the phylum known as endodyogeny [14] as a model. We find that in *T. gondii* striated fiber assemblin (SFA) proteins, whose orthologs are found in the rootlet associated with flagellar basal bodies of single celled algae, assemble into a highly dynamic fiber during cell division. The SFA fiber links the centrosome and daughter MTOC, and ablation of SFA by conditional knock out results in multinucleated cells that fail to initiate the formation of daughter cells.

## Results

During cell division *T. gondii* assembles two daughter cells with a complex microtubular cytoskeleton and secretory apparatus within the mother cell (Figure 1A). This process has to be coordinated with mitosis and organelle segregation to ensure that the emerging daughter cells not only are competent to invade new host cells, but also carry the genetic and metabolic machinery required to propagate. It is not well understood how each daughter cell inherits a complete set of essential organelles. SFA is the main component of striated rootlets associated with basal bodies in green algae [15]–[17]. In previous work, Lechtreck and colleagues identified genes encoding homologs of SFA in Apicomplexa, including *T. gondii* [18]. This finding was surprising because, with the exception of the male gamete, Apicomplexa lack flagella. Nonetheless, antibodies raised against SFA from the green alga *Spermatozopsis similis* revealed a spot in proximity of the centrosome in *T. gondii* [18]. Transcription of the *T. gondii* SFA genes is cell cycle dependent with peak expression coinciding with DNA synthesis and mitosis (Figure 1B) [19],[20]. We therefore hypothesized that SFAs may function during division of apicomplexan parasites.

To define the function of SFA proteins in *T. gondii*, we first determined their localization. We focused on TgSFA2 and TgSFA3, two proteins that are expressed in the tachyzoite stage maintained in tissue culture. We engineered parasites in which the native TgSFA2 is tagged with a triple hemagglutinin (3×HA) at its C terminus. Southern blot analysis with a probe complementary to the 3′ end of TgSFA2 confirmed the insertion of the tag into the locus (Figure S1A). Western blot showed a single band of the mass predicted for TgSFA2-3HA (30 kDa) to be recognized by anti-HA antibodies (Figure 1D). To study TgSFA3, we expressed the gene in *Escherichia coli*, and raised antibodies against the recombinant protein. Independently, we also generated a strain in which TgSFA3 is endogenously tagged with yellow fluorescent protein (YFP) at its C terminus. A Western blot with anti-GFP antibodies showed a fusion protein of expected mass in the TgSFA3-YFP cell line, but not in the parental cell line (Figure 1D). This band is also recognized by the anti-SFA3 antibody, which in wild-type parasites recognizes native TgSFA3 (35 kDa) and the recombinant protein in bacterial lysates (rSFA3, Figure 1D). We next performed immunofluorescence assays (IFAs) on TgSFA2-HA parasites using HA and anti-SFA3 antibodies to detect both proteins simultaneously. TgSFA2 and TgSFA3 largely co-localize and both reveal two short fiber-like structures per parasite cell (Figure 1E).

**Figure 2:**
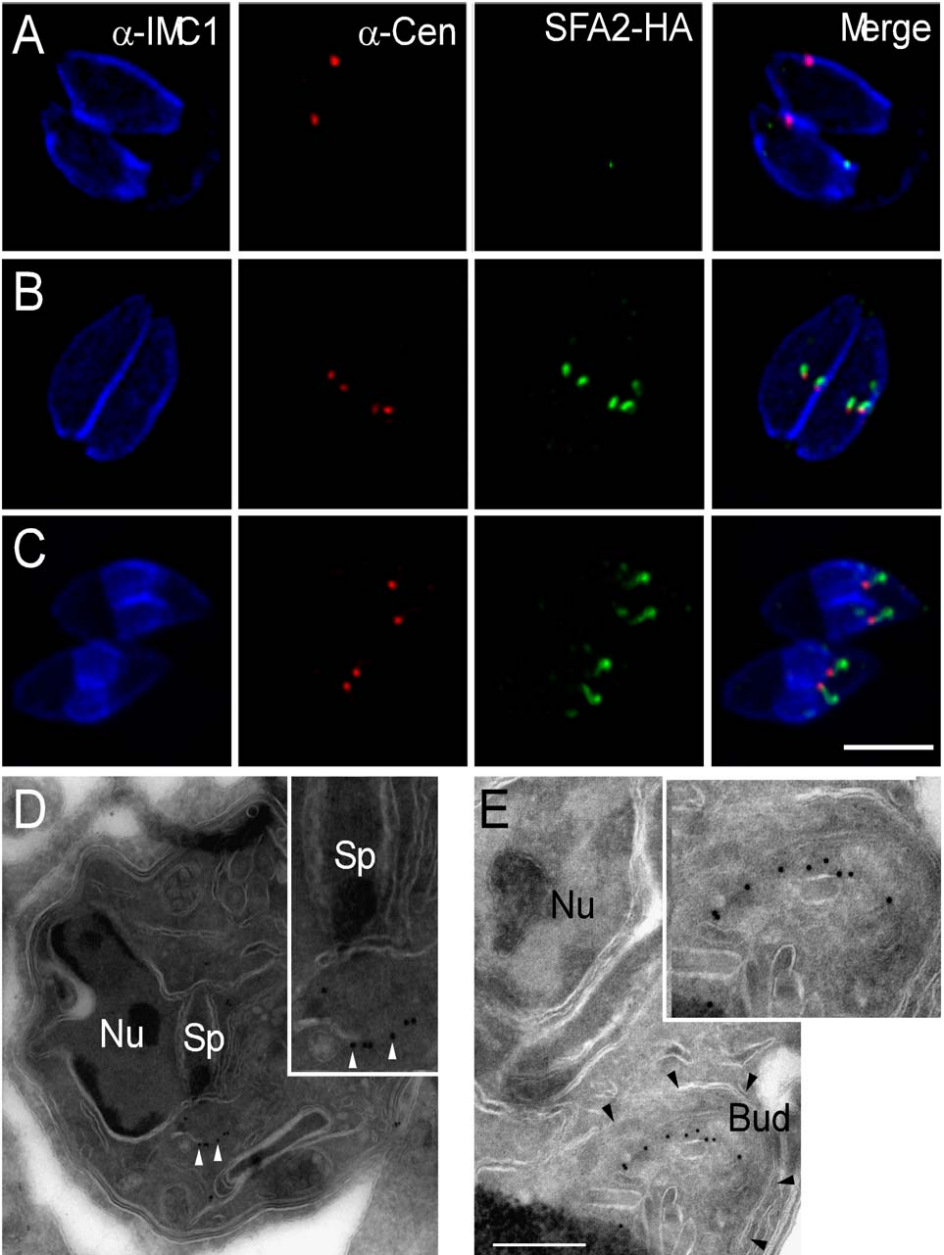
*T. gondii* SFAs are expressed only during cell division. (A–C) IFAs of SFA2-HA. Parasites were labeled with anti-HA (green), in combination with anti-IMC1 (blue) and anti-centrin (red). IMC1 labels the IMC in both the mother and the daughter cell and centrin is a marker of the centrosome. Robust HA staining is only apparent in parasites with duplicated centrosomes. Scale bar = 5 µm. (D and E) SFA2-HA parasites were fixed, cryo-sectioned, and probed with anti-HA antibody, followed by incubation with gold conjugated protein A. (D) The spindle (Sp) is visible in an invagination of the envelope of the nucleus (Nu), note vertical white lines representing spindle microtubules. Gold particles are highlighted by white arrowheads. (E) IMC outlining a daughter bud is highlighted by black arrowheads. Scale bar = 500 nM. Insets show further enlargements for detail.

While observing endogenously tagged TgSFA2 or the labeling by the TgSFA3 antibody by immunofluorescence we noticed that only a fraction of the parasites showed staining. At a given time 24% of the parasites express TgSFA2, while 76% do not (Figure 1C). These percentages closely match those previously reported for interphase and dividing parasites in asynchronous populations of *T. gondii* [21]. Next, we co-stained cells for TgSFA2-HA and with antibodies that detect markers of *T. gondii* cell cycle progression including centrin and IMC1. Centrin is a marker of the centrosome (Figure 2A–2C, red), IMC1 (blue) is part of the cytoskeleton of the IMC of the pellicle and outlines the mother cell as well as the forming daughters [22],[23]. Most parasites appear to be in G1 or early S phase, as determined by the presence of a single centrosome per parasite. Consistent with our prediction, no SFA labeling is discernible in these interphase cells. After centrosome duplication, SFA labeling becomes apparent as small punctuate structures that are very close to or overlap with centrosome labeling. In parasites that show anti-IMC1 stained daughter buds, TgSFA2 labels a long structure, extending away from the centrosome (Figure 2C). Notably, the SFA fiber is arched with a spiraling hook shape at its distal end. This pattern is also apparent in immuno-gold labeled cryosections of SFA2-HA parasites. Figure 2D shows the intra-nuclear mitotic spindle of *T. gondii*, an early step in the budding process [2],[14]. A short series of gold particles is visible at the bottom spindle pole. In parasites progressed further in division, gold particles form an arched line that climbs into the apical end of the daughter bud (Figure 2E). We conclude that SFA2 and 3 form a structure early in mitosis, that extends into a fiber during budding, but is absent in interphase.

**Figure 3:**
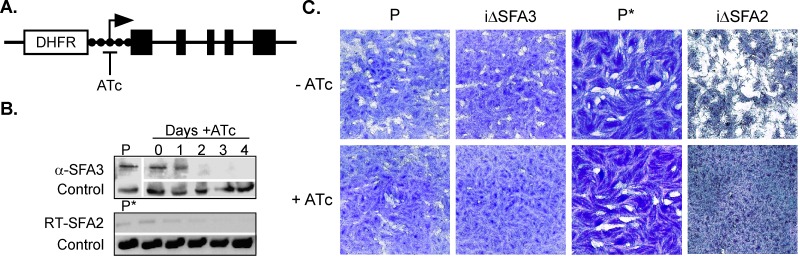
TgSFA2 and TgSFA3 are required for parasite growth. (A) Simplified schematic of iΔSFA3 promoter insertion mutant (see supplemental Figure S2 for further detail). Promoter activity is inhibited by addition of ATc to the growth medium. (B) Top, Western blot using anti-SFA3 antibodies to measure SFA3 in the iΔSFA3 strain upon ATc treatment (P, parent; tubulin is shown as a loading control). Bottom, reverse transcriptase PCR analysis of SFA2 transcript in the iΔSFA2 strain upon ATc treatment (RT-PCR of TGGT1_021600 transcript is shown as control). Note that the expression of both targeted genes is susceptible to ATc. (C) Plaque assay measuring growth of mutants and parental strains in the presence (+) or absence of ATc (−). Note that both mutant strains fail to form plaques (clearings) in a monolayer of fibroblast in the presence of ATc in the medium. The parental strains and untreated cultures of all strains are shown for comparison. The asterisk indicates that the parental strains (P and P*) of the mutants are distinct (see supplementary Figure S2).

To test whether SFA proteins have a functional role in parasite division we generated mutants in which their expression can be manipulated (Figure 3A). We constructed a strain, iΔSFA3, in which the native promoter of SFA3 is replaced by a tetracycline-regulatable promoter [24],[25]. The targeting construct was derived from a cosmid clone carrying the SFA3 locus by recombineering [24],[26]. A corresponding strain for SFA2 (iΔSFA2) was also made. In this case we used a plasmid that was designed to introduce both a regulatable promoter and a transactivator into the locus (both strategies and the specific parental strains used are described in detail in the Materials and Methods section and Figure S2). Disruptions of the targeted loci were confirmed by PCR (Figure S2B and S2D). Mutant parasites were cultured in the presence of anhydrotetracycline (ATc) and targeted protein levels were measured by Western blots using anti-SFA3 antibodies or by reverse transcription-PCR for the SFA2 mRNA. We noted a marked decrease in the levels of the targeted SFA proteins or mRNAs after 2 d of ATc treatment (Figure 3B). In both mutant strains parasite growth was severely impaired in the presence of ATc, as documented by their inability to form plaques in a fibroblast monolayer (Figure 3C, note that plaque formation of the parental strains is not affected by ATc). We conclude that SFA2 and SFA3 are non-redundant and both are required for parasite viability.

**Figure 4:**
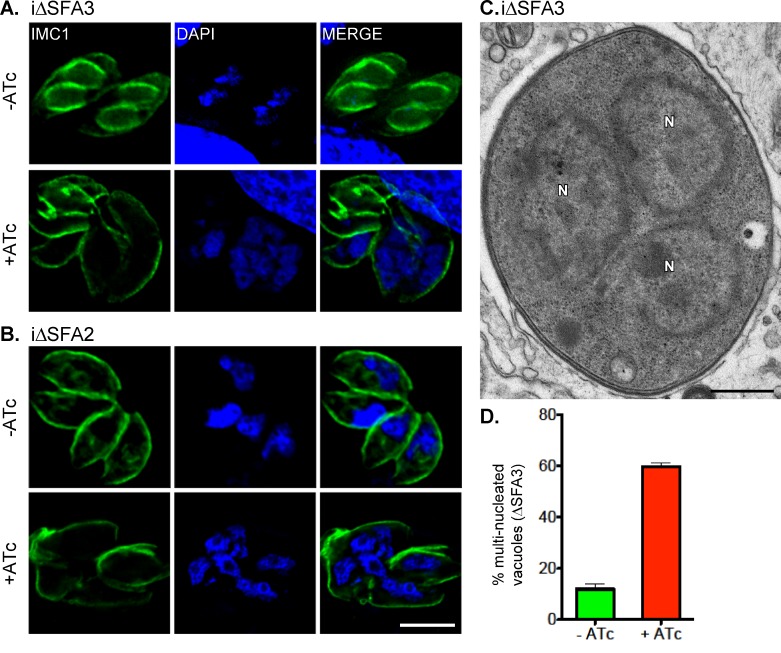
Parasites lacking TgSFA2 or TgSFA3 show a pronounced cell division defect. IFAs of cells infected with iΔSFA3 (A) or iΔSFA2 (B) mutant parasites cultured for 48 h in presence or absence of ATc prior to fixation. Note that both mutants accumulate multiple nuclei (blue), and fail to form proper buds (IMC1, green) under knock down conditions. Untreated controls divide normally. (C) Transmission electron micrograph of iΔSFA3 parasites grown in presence of ATc for 48 h. This section through a cell shows three nuclei (N; scale bar = 500 nm). (D) Multi-nucleated (≥2) parasites were quantified for iΔSFA2 parasites (48 h ± ATc) using DAPI and IMC1 staining. 30 randomly chosen fields were counted and the percentage of vacuoles containing parasites with multiple nuclei is graphed. Error bars represent standard deviation (*n* = 3).

**Figure 5:**
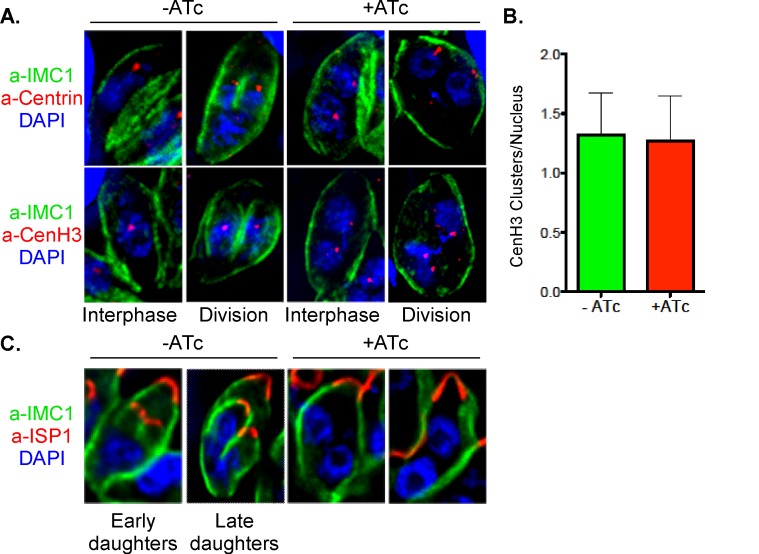
Parasite lacking SFA go through mitosis normally but fail to form daughter cells. (A) iΔSFA2 parasites grown in the presence or absence of ATc for 24 h were labeled with anti-IMC1 (green) and DAPI (blue) anti-centrin (red, centrosomes, upper panels) or anti-CenH3 (red, centromeres, lower panels). Representative examples are shown for cells in interphase or division. Interphase nuclei associate with a single centrosome, while larger 2N nuclei associate with two. This is unchanged by ATc treatment. Interphase nuclei contain a haploid genome and exhibit one CenH3 dot representing a cluster of the centromeres of all 14 chromosomes bundled in the nucleus in close proximity to the centrosome (see [9]). Dividing nuclei exhibit two CenH3 dots representing duplicated chromosomes. Again, this labeling pattern is not affected in the mutant when judged on a per nucleus basis. Note though that in both cases the ATc-treated cell is already tetraploid with no sign of cytokinesis. (B) The number of CenH3 dots per nucleus was quantified in IFA experiments for untreated or ATc-treated iΔSFA2 parasites. The number of CenH3 dots, representing the number of chromosome sets per nucleus, is graphed, error bars show standard deviation (>50 parasite vacuole per experiment counted, *n* = 3). (C) Immunofluorescence of iΔSFA2 parasites after 48 h ATc treatment showing IMC1 (green), DAPI (blue), and anti-ISP1 (red). ISP1 labels the apical cap of the IMC of both mother and daughter cells and is an early marker of budding [27]. ISP1 staining for daughter cells is absent in the ATc-treated mutant. Note that the nuclei have completed mitosis in these cells and compare to a similar stage shown for untreated parasite.

We hypothesized that the growth arrest of mutants deficient in SFAs is caused by defects in cell division. We cultured the mutant parasites for 24 and 48 h in ATc and stained using anti-IMC1 antibody to outline cells and DAPI to visualize nuclei. As shown in Figure 4A and 4B, for both mutants ATc treatment resulted in excessively large cells bearing multiple nuclei. We quantified this phenotype in the iΔSFA3 strain (Figure 4D), 59% of parasite cells are multinucleated (≥2 nuclei per cell) after 24 h of ATc treatment. Parasites with numerous apparently normal nuclei are also readily observed by electron microscopy (Figure 4C). To evaluate nuclear division and chromosome segregation more rigorously we stained mutants with a monoclonal antibody that we developed against the *T. gondii* centromeric histone variant 3 (CenH3). In Apicomplexans, centromeres are sequestered at the nuclear envelope in a centrosome-dependent manner and haploid and diploid nuclei have a single or duplicated CenH3 spot, respectively [9],[10]. We quantified the nuclear ploidy and note that ATc-treated mutants and controls are indistinguishable (Figure 5B). Moreover, we observed that every nucleus is associated with one or two centrosomes and that the centromere–centrosome association appears undisturbed (Figure 5A).

We next monitored daughter cell formation. Normally, IMC1 outlines the pellicle of both the mother and daughter cell (see Figure 2A and 2B). Strikingly, mutant parasites containing multiple nuclei showed aberrant or no daughter cells when stained with anti-IMC1 (Figure 4A). To test whether SFAs are required for the initiation or elaboration of daughters we stained mutants for the early marker of budding IMC subcompartment protein 1 (ISP1). ISP1 labels the apical cap of the IMC and can be detected prior to IMC1 [27]. In ATc-treated parasites ISP1 is visible in the mother cell pellicle but no daughter structures are discernible (Figure 5C). Taken together, these results suggest that loss of SFA proteins does not affect centrosome duplication and mitosis but severely impedes budding.

**Figure 6:**
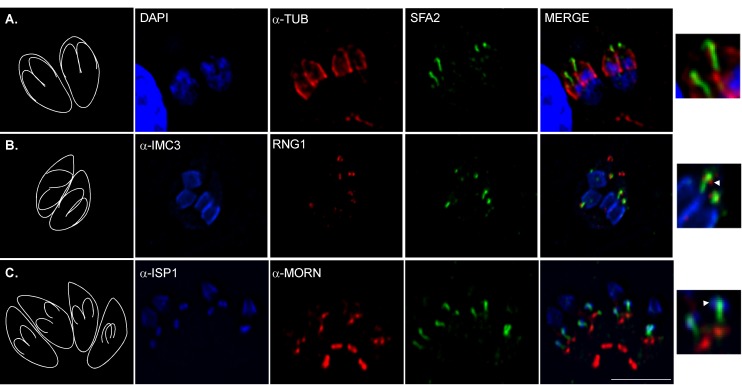
The SFA fiber extends in the apex of the forming daughter. IFAs showing SFA2-HA stained with anti-HA (green) in combination with markers for daughter bud. (A) anti-tubulin (red) labels the sub-pellicular microtubules. SFA2 staining extends beyond the sub-pellicular microtubules and into the conoid. (B) Anti-IMC3 (blue) labels the IMC of emerging daughter cells and is shown as a reference for the position of daughter cells. RNG1-YFP was detected using anti-GFP antibodies (red). RNG1 is a marker of the conoid [29]. The SFA2-HA signal extends into and slightly beyond the ring of RNG1 staining. (C) anti-ISP1 (blue) labels the apical cap of the IMC. Anti-Morn1 (red) labels the basal complex of both the mother's and the daughter's IMC, as well as, a nuclear structure in immediate proximity of the centrosome [30],[31]. The SFA2-HA signal spans from the centrosome region to the apex of the daughter bud. Scale bars = 5 µm.

**Figure 7:**
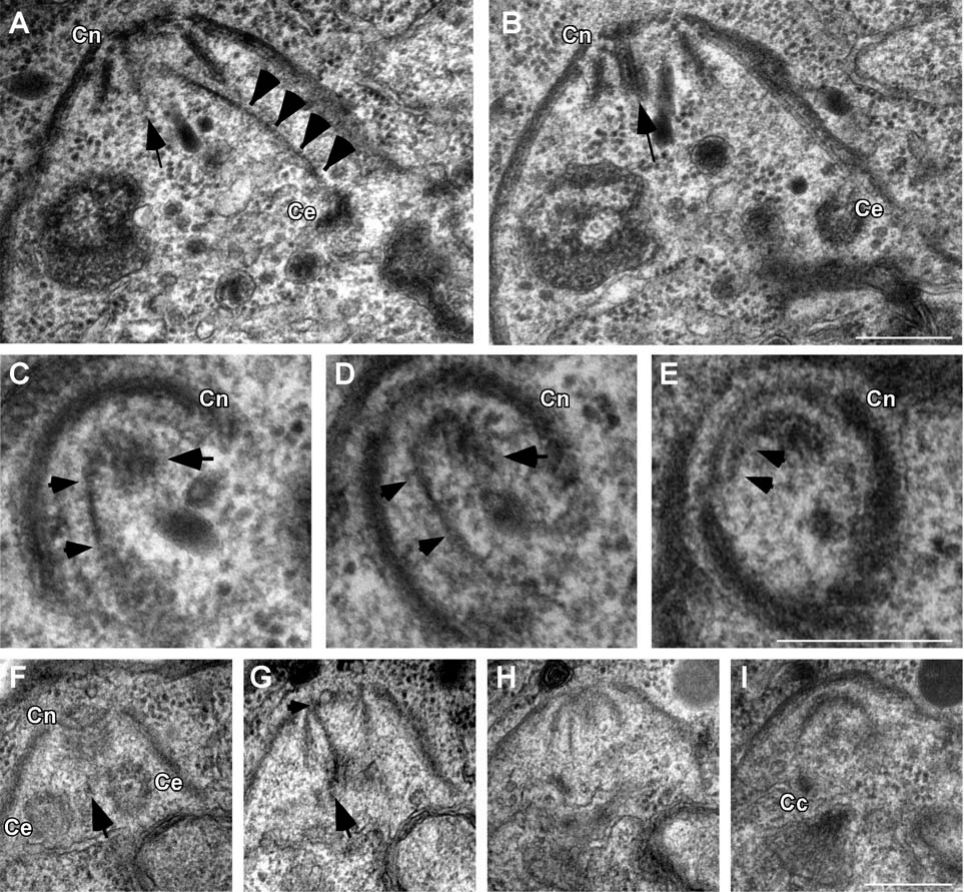
A fiber links the centrosome and the apical complex of the daughter bud. Transmission electron micrographs of serial sections of dividing wild-type parasites. (A and B) Two consecutive sections through a daughter cell are shown. An electron dense structure is highlighted in (A) (arrowheads, note some striation in this section, in particular toward the conoid end) emanating from close to one centriole (Ce; B) and reaching up to the conoid of the daughter cell (Cn; A and B). The structure ends in close proximity of the central pair of microtubules within the conoid (arrow; A and B). (C–E) Three consecutive sections perpendicular to the conoid and the orientation of the parasite depicted in (A and B) are shown. A bent electron dense structure (arrowheads) runs within the conoid (Cn) towards the apical ring. The end of this structure appears in contact with the conoid-associated microtubule pair (arrow, also see Figure 10 for a schematic outline). (F–I) Four consecutive sections through an early daughter cell. Both parallel centrioles (Ce) of the centrosome can be seen in (F). A fiber is visible reaching into the conoid and touching the apical ring (Cn; F–H). The fiber emerges between the centrioles (arrow; F and G). The last section shows intra-nuclear microtubules as part of the centrocone (CC, a nuclear envelope structure associated with centromere organization in Apicomplexa); these link the kinetochores of the chromosomes to the centrosome. Scale bars = 250 nM.

To better understand the mechanistic basis of the mutants' inability to bud, we monitored the localization of SFAs in relation to structures important for daughter cell assembly throughout division. Daughter cells are formed on a stereotypic scaffold of 22 sub-pellicular microtubules that arise from a circular apical organizing center, the apical polar ring [28]. In *Toxoplasma* this structure also includes the conoid, a motile structure thought to be involved in host cell invasion [5],[6]. We examined the localization of the SFA fiber relative to that of alpha-tubulin, Ring1 (RNG1), a component of the apical polar ring [29] and ISP1 [27]. We observed that the apical end of the SFA fiber consistently extends beyond the end of tubulin staining corresponding to the sub-pellicular microtubules (Figure 6A) and terminates at the apex of developing daughters, extending through the RNG1 staining and to the very tip of the ISP1 staining (Figure 6B and 6C). To unravel this complex architecture we imaged serial sections of dividing parasites by transmission electron microscopy (TEM). Figure 7A and 7B shows two consecutive sections through a daughter bud in which the conoid (Cn) and centrosome (Ce) are clearly identifiable. Spanning the area between them is an arching electron dense fiber (black arrowheads). The fiber terminates at a pair of microtubules that extend through the center of the conoid (arrow, [3]). Figure 7C–7E show a series of perpendicular sections through the conoid of a daughter cell. An electron dense fiber (arrowheads) curls up through the conoid coming into close contact with the apical rim of the structure and ending in the proximity of the central microtubule pair (arrow). A series of sections through a very early daughter bud shows the fiber to be already present at this stage (Figure 7F–7I). Note that it again makes contact with the apical rim of the conoid (Figure 7G, arrowhead) and that it emerges from in between the two centrioles of the centrosome (Figure 7F and 7G, arrow). Our light and electron microscopic observations suggest that the SFA fiber physically connects the centrosome to the tip of the forming daughter cell.

**Figure 8:**
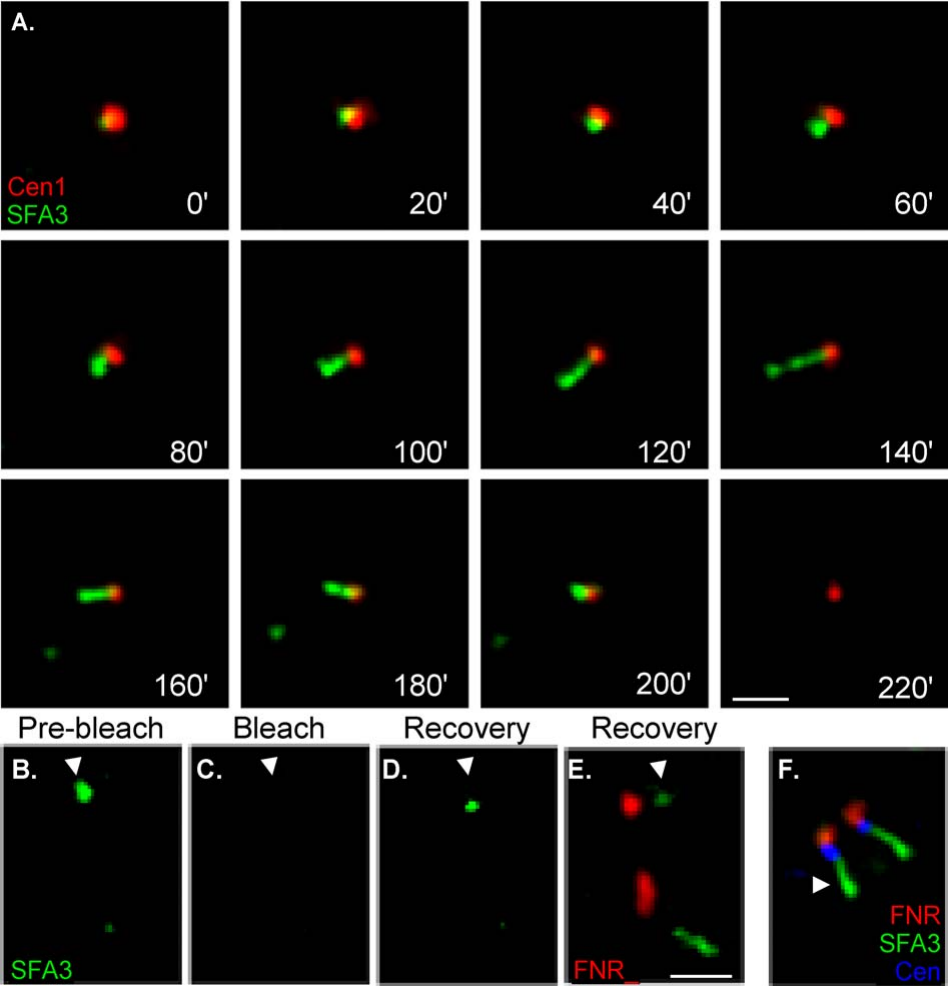
The SFA fiber grows in a polar fashion away from the centrosome. (A) Time-lapse imaging of SFA3-YFP parasites expressing Centrin1-RFP. Images were taken every 10 min for 220 min. Note that SFA-YFP forms a centrosome associated spot that extends away from the centrosome (min 60–120). The fiber breaks at its distal end (min 140–160) and then shortens in reverse order. See Video S2 for an animated version. (B–E) Photo bleaching assay of TgSFA3-YFP parasites expressing FNR-RFP (Ferredoxin/NADPH Reductase-RFP). (B) The fiber in the focal plane (arrowhead) was 0.45 µM prior to bleaching. (C) The target fiber was bleached using a 488-nm laser and is no longer visible after the laser pulse. (D) Images of the photo-bleached fiber were taken after 1 h to monitor recovery of the YFP signal. YFP labeling of the fiber can again be appreciated spanning 0.35 µM. (E) Image showing the YFP labeled fiber after bleaching, its sister unbleached fiber, and FNR-RFP for reference. The right most fiber (unbleached) spans 0.85 µM. The bleached fiber appears shorter and that (new) labeling appears proximal to the FNR-RFP signal. (F) IFA showing the close proximity and relative orientation of apicoplast (FNR-RFP, red), centrosome (blue), and SFA fiber (green). The apicoplast (FNR-RFP) associates with the centrosome during division [12], and was used as a marker for the position of the centrosome. Scale bar = 1 µm.

To visualize the dynamic development of the SFA fiber we inserted a YFP coding sequence into the genomic locus of SFA3 creating a C-terminal fusion protein. We time lapse imaged the SFA3-YFP strain and determined that SFA3 is visible for 2 h and 20 min (*n*>5), a time frame consistent with the duration of mitosis in *T. gondii* under imaging conditions [21]. Moreover, we observed that the SFA structure is dynamic and its morphology changes with time (Video S1). In order to have a spatial reference for the transition events of the SFA fiber we imaged SFA3-YFP in combination with Centrin1 fused to red fluorescent protein (RFP). This allowed us to concurrently monitor the position of the SFA fiber and the centrosome [30],[31]. In time-lapse imaging the YFP signal appears right on the centrosome (Figure 8A; Video S2). The fiber then elongates away from the centrosome. When the fiber reaches about half of its final length a “hook”-like shape at the tip away from the centrosome can be resolved (Figure 8A, 100′). The fiber reaches a maximum length of about 1 µm, at which point it appears to break close to its distal end (Figure 8A, 140′–160′). This leaves a small dot (presumably associated with the tip of the daughter cell). The longer centrosome associated portion shortens from the distal end and finally, SFA3-YFP is no longer detectable leaving only the centrosome visible (Figure 8A, 200′).

Polarized polymerization of subunits similar to microtubules or actin filaments could be a model for the growths of the SFA. To test this idea and to determine the direction of fiber extension we used fluorescence recovery after photo-bleaching. We chose SFA3-YFP parasites exhibiting two fibers of medium length (0.45 µm) and selectively bleached one of the two fibers using a diffraction limited 488 nm laser spot (Figure 8C). Figure 8B shows the target fiber prior to bleaching, note that the second fiber is not in the same focal plane as the target fiber and does not appear in this series of images. Figure 8C shows that after the laser pulse, the target fiber is no longer visible. We monitored the fluorescence of the bleached fiber after 1 h, and found reappearance of the YFP signal (Figure 8D). While the unbleached fiber had practically doubled in size to 0.85 µm, the YFP signal on the bleached fiber was only 0.35 µm (Figure 8D and 8E). For reference we also imaged the apicoplast labeled with ferredoxin/NADPH reductase-RFP (FNR-RFP). During division the apicoplast shows close apposition to the centrosome (see Figure 8F) [12]. When compared to the control fiber SFA3-YFP labeling of the bleached fiber appeared to be polar and proximal to the apicoplast. Thus, it appears that the fiber grows out by polymerization and that the new subunits are added at the end proximal to the centrosome which could be considered the “plus” end of the fiber. We note that we currently do not have a suitable probe to observe the bleached segment of the fiber, and thus cannot measure its entire length. We therefore cannot formally exclude proximal labeling due to laser induced stunting or breakage of the fiber.

**Figure 9:**
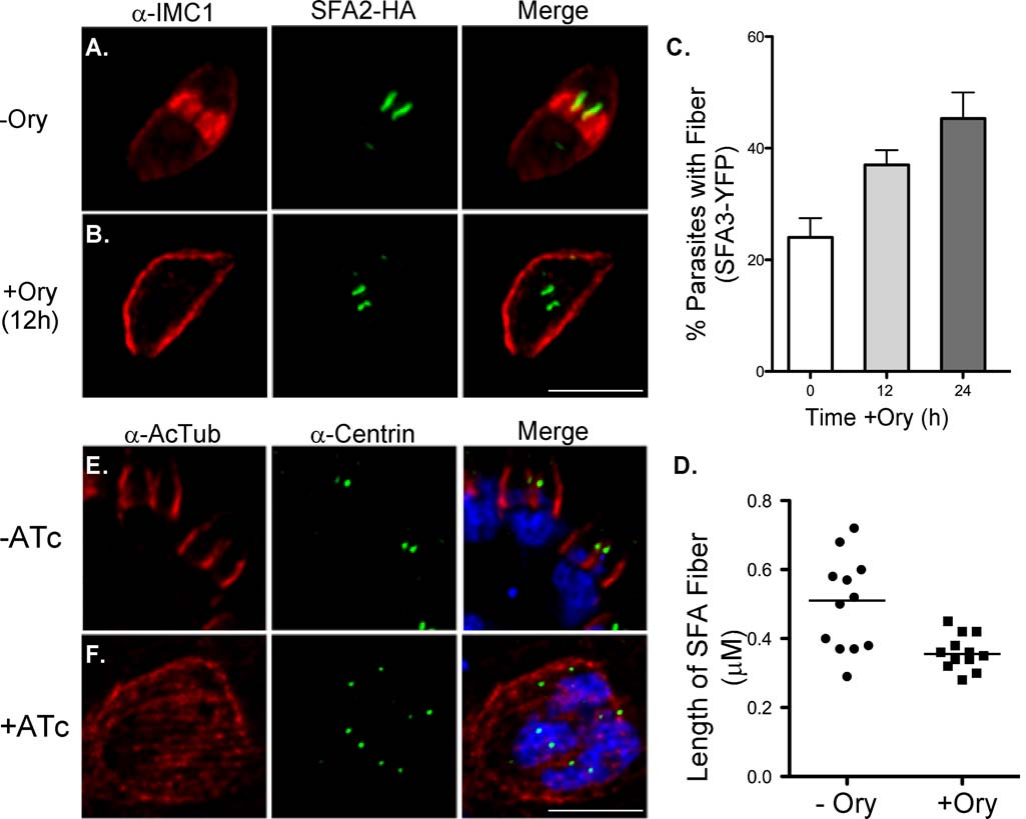
Daughter microtubules depend on SFA fiber, but the fiber does not depend on microtubules. (A and B) Immunofluorescence of SFA2-HA parasites treated with oryzalin, a microtubule-disrupting agent that prevents formation of new microtubules in daughter cells, and that has a more moderate effect on existing stable microtubules in the mother [34],[35]. Note that after 12 h of treatment parasite cells fail to assemble buds (B) and compare to the matched control (A). SFA2-HA fibers are nonetheless detected. Note that in untreated parasites, daughter cells are normally detectable when similar fibers are observed. (C) SFA3-YFP parasites were treated with oryzalin and scored for the presence of SFA3-YFP after 0, 12, or 24 h of treatment. Note that treated parasites accumulate fibers. (D) Fiber length was measured in control parasites and parasites treated with oryzalin for 24 h. Data points reflect the mean fiber length per field of view scored (*n* = 18–81 fiber/field, four fields for each of three independent repeats). Fibers of treated parasites are overall shorter and more uniformly distributed in size. iΔSFA2 parasites were grown in the absence (E) or presence of ATc (F) and stained for acetylated tubulin (red), centrin (green), and DAPI (blue). The anti-acetylated tubulin antibody labels daughter buds strongly (as do antibodies to unmodified tubulin) in untreated mutants. ATc-treated iΔSFA2 parasites exhibit acetyl-tubulin staining exclusively in the mother cell cytoskeleton (note that two mutant cells are shown with microtubules encaging each entire cell; the cells are abnormally large due to the block in budding). No daughter microtubular-skeletons are discernible in these cells despite the fact that each cell shown has two nuclei, both of which are entering mitosis as indicated by the duplicated centrosomes. Scale bars = 5 µm.

Rootlet fibers are typically found in intimate contact with basal bodies or microtubules [15],[32]. Our observations are consistent with a polar SFA fiber that places and potentially governs the formation of the daughter cell and/or its MTOC. Alternatively, newly formed microtubules, for example, the central pair, could be recruiting the fiber to the MTOC, tethering the daughter in a secondary fashion. To distinguish between these two alternatives we tested whether daughter cell microtubules are required for SFA fiber formation or vice versa. We stained microtubules in the iΔSFA3 mutant using different tubulin antibodies. In Figure 9E, 9F. we show antibody 6–11B directed against acetylated alpha tubulin as this antibody recognized daughter cell microtubules particularly well [33]. In mutants treated with ATc for 48 h no daughter microtubules are detectable. Note that these particular cells (Figure 9F) are already multinucleated and undergoing another round of mitosis as indicated by the presence of two centrosomes per nucleus. Microtubules of the mother cell are readily detected. Conversely we treated SFA3-YFP or SFA2-HA parasites with 2.5 µM of the microtubule disrupting agent oryzalin [34]. As previously reported [35] oryzalin-treated parasites fail to produce daughter cells; note the lack of daughter IMC1 staining in Figure 9B. However, in these parasites SFA fibers are still detected (Figure 9A and 9B, green). In fact fibers are noticeably more abundant; 60% of vacuoles exhibit SFA fibers after 24 h of oryzalin treatment while only 25% of the control parasites do (Figure 9C). We further noticed that in oryzalin-treated parasites SFA fibers remain shorter and show a more uniform size distribution when compared with untreated parasites (Figure 9D). We conclude that daughter cell microtubules are not required for SFA fiber formation but that microtubule elongation may be required for the fiber to extend to its full length, break, and disappear. Alternatively, fiber elongation might require licensing by a checkpoint controlled by daughter cell growth.

## Discussion

**Figure 10:**
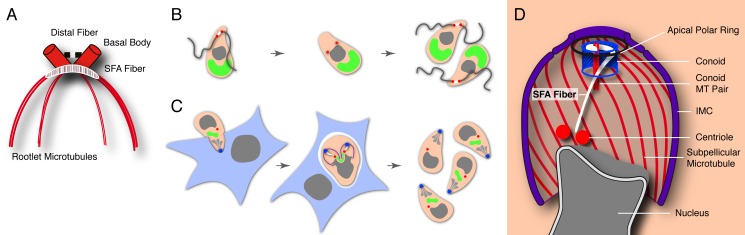
Is the apical host cell invasion complex derived from the flagellum of the algal ancestor? (**A**) Schematic of the flagellar rootlet system of *Chlamydomonas* (simplified after [64]). The two flagellar basal bodies are coordinated by rootlet fibers (only SFA and distal fibers are shown here) and bundles of rootlet microtubules (two or four microtubules each) (**B**). Schematic outline of cell division in the hypothetical flagellated algal ancestor of Apicomplexa. Basal bodies of the flagella also serve to organize the mitotic spindle (flagella are resorbed or shed during mitosis in some flagellated algae, note that number and behavior of flagella in the apicomplexan ancestor is hypothetical). Rootlet fibers (white) may have additional roles in division [32],[37],[38]. (**C**) Apicoplast, green; nucleus, grey; SFA fiber, white; basal body/centrosome, red; conoid, blue, rhoptries (secretory component of the apical invasion apparatus), light grey. Apicomplexans are intracellular parasites and have lost flagella in most stages. SFA rootlet fiber is only expressed during division and coordinates the centrosome with the MTOC of the daughter bud. This suggests that the system that controlled the positioning and assembly of flagella in the ancestor now organizes the assembly of the apical host cell invasion complex. (**D**) Schematic of the SFA fiber and its relationship to other cellular structures during *T. gondii* cell division (only a single daughter bud is shown for simplicity).

Flagella provide motility and sensory functions to a large variety of single and multicellular eukaryotes. They are anchored in the cell by the basal body [36]. Flagellar basal bodies are embedded within a complex cytoskeletal system known as the flagellar rootlet system or basal body cage, which has been studied in most detail in the green alga *Chlamydomonas reinhardtii* [32]. There is evidence that these structures not only position the flagella but also define cellular axes of symmetry and asymmetry [37],[38]. The rootlet is composed of several types of biochemically and structurally distinct fibers some of which are made of microtubules. In *Chlamydomonas*, centrin-based fibers (also known as contractile fibers) interconnect basal bodies and connect the basal bodies to the nucleus [39]. Sinister fibers, first described in *S. similis*, connect the basal bodies to two of the four microtubules of the flagellar rootlet and to cytoplasmic microtubules [40]. Striated fibers are made up from a single SFA protein and run along microtubules, emerging close to basal bodies in post mitotic cells, and are thought to guide and stabilize their associated microtubules (Figure 10A) [17],[18]. It has been proposed that the mechanism by which SFA binds microtubules is related to the structure of a rod domain found in the protein which consists of 29 amino acid repeats. The periodicity of this repeat confers the characteristic striation pattern found in SMAFs, and also fits the spacing between tubulin subunits in microtubules [18]. Striated fibers are also found in association with basal bodies in mammalian cells (e.g., various receptor cells), but the proteins isolated from these fibers do not appear to be homologous to SFA proteins [41].

In this study we show that SFAs play a critical role in the cell division of the apicomplexan parasite *T. gondii*. We identified a fiber that is made up of at least two proteins, TgSFA2 and 3. This structure becomes apparent as soon as the centrosome is duplicated; it emerges from in between the two centrioles and grows away from the centrosome. Its distal end is intimately associated with the apical tip of the daughter cell (Figure 10D summarizes our current ultrastructural understanding). Interestingly, the SFA fiber is not only a tether between centrosome and the daughter; it is required for daughter assembly. In conditional mutants lacking the SFA fiber we do not detect daughter buds even using the earliest markers available. The “birth” of the daughter is the establishment of the apical MTOC. We propose that the distal end of the SFA fiber initiates and thus positions the daughter MTOC. Could the fiber itself be an MTOC? Several studies have demonstrated the ability of algal rootlet complexes to initiate microtubule assembly in vivo and in vitro [42],. It is tempting to note the peculiar shape of the end of the *Toxoplasma* fiber in the context of the circular MTOC found in these organisms and to speculate that the structure of the fiber may template the microtubule arrangement of the daughter pellicle. Further biochemical work is required to test this hypothesis in vitro. *Chlamydomonas* SFA has been demonstrated to have the intrinsic ability to self-assemble and self-organize; recombinant SFA forms striated fibers in vitro [44].

Why does a cell in a stage lacking flagella use a budding system that depends on elements of the flagellar rootlet? We believe this to be a consequence of the evolutionary history of Apicomplexa. The assembly of the flagellum and the mitotic spindle share deep evolutionary roots. The centrioles, which are at the core of many, but not all mitotic spindle poles, are homologous to the basal bodies [36]. In fact, in many cells the very same structure performs both functions. In *Chlamydomonas*, during interphase two closely apposed basal bodies organize the organisms' two flagella [32]. These flagella are resorbed upon entry into cell division and the basal bodies become associated with the poles of the mitotic spindle. Following division both daughters assemble a transition zone onto the centrioles and reform flagella. The rootlet (shown schematically in white in Figure 10B) appears to be important in both roles setting up division and symmetry planes and positioning the centrosome and the flagellum relative to each other as the cells move through their replicative cycle [37],[38]. Apicomplexa are believed to have an evolutionary past as photosynthetic aquatic algae. They are part of the Chromalveolata, a large branch of the eukaryotic tree of life that emerged from the endosymbiosis between a flagellated protist and a red alga [45]. The most conspicuous holdover of this past is a chloroplast-like organelle [46]. We hypothesize that the ancestors of Apicomplexa, as their present day kin likely depended on the rootlet to organize the relationship of their flagellar and mitotic MTOCs. As they adapted to intracellular parasitism they developed specialized cytoskeletal and secretory organelles that allow them to attack other cells [1]. Some precursors of these organelles are found in closely related flagellated protists that are fully or partially symbiotic, predatory or parasitic—in all these cases the organelles are found in close proximity to the flagellar basal body [47]–[49]. We propose that Apicomplexa subsequently abandoned the flagellum for most stages yet retained the organizing principle of the MTOC. Instead of ensuring that daughter cells have appropriate numbers of flagella the system now measures out and positions the apical invasion complexes (Figure 10C). Overall this suggests that elements of the invasion apparatus may be derived from flagella- or flagellum-associated structures.

In combination with the recently described tethering of the nuclear genome and other organelles [9],[10],[12], a remarkably hard-wired model for the assembly of infective parasite stages emerges. The role of the SFA fiber is crucial in this context, a self-organizing polar fiber that will initiate a daughter in the proximity of each centrosome once its components are expressed. The elegance of this mechanism is its scalability and independence of ploidy. It satisfyingly explains how *T. gondii* can form two daughters per round of budding, while *P. falciparum* forms ten to 20 in the red cell, and many thousands during liver cell infection. Direct evidence for the control of zoite formation by the flagellar rootlet is currently limited to the experiments with *T. gondii* presented in this study. However, we note that SFA homologs are encoded in the genomes of all apicomplexans for which sequence is available (and many other chromalveolates [50]). Furthermore electron dense structures comparable to those identified as SFA fibers in this study have been observed in previous ultrastructrual reports in *Eimeria* and *Plasmodium* [51]–[53]. Many mechanistic questions remain, some of them directed towards the relationship between the structure of the fiber and its function. There are also intriguing problems associated with the spatial and temporal control of initiation and breakdown of the structure, and how they are integrated into the parasites mechanisms of cell cycle control, which remain to be deciphered.

## Materials and Methods

### Construction of Tagged Reporter Parasites

*T. gondii* RH strain parasites were maintained by serial passage in human foreskin fibroblast (HFF) cells and genetically manipulated as previously described [54]. To tag the genomic locus of TgSFA2 (GenBank accession XM_002367757) with a 3×HA tag, 585 bp of the open reading frame ending before the stop codon was amplified from the *T. gondii* genomic DNA. All primer sequences used are shown in Table S1. Similarly, 3,000 bp upstream of the stop codon of XM_002370621.1 were amplified to tag TgSFA3 with YFP. These amplicons were cloned via ligation-independent cloning (LIC) [55] into the pLIC-HA-CAT or pLIC-YFP-DHFR vector, respectively, to create in-frame fusions [56]. Transgenic clones were established by transfection of ΔKu80 parasites and chloramphenicol or pyrimethamine selection, respectively, as previously described [56]. Integration was confirmed by PCR or Southern blotting as previously described [57]. A probe complementary to the 3′ region of the SFA2 gene was amplified by PCR. Cen1-RFP [30] was introduced into SFA3-YFP parasites by transient transfection. FNR-RFP [57] was transfected into SFA3-YFP parasites and stable transgenics were isolated by fluorescence activated cell sorting [54].

### Construction of Conditional SFA2 and SFA3 Knock Out Parasites

To target SFA2, 1,500 bp immediately up- and downstream of the start codon were amplified and introduced into vector piKO in order to flank an HXGPRT selectable marker [58], a transactivator (TaTi) and the tetracycline regulatable T7S1 promoter (this plasmid was a kind gift of Dominique Soldati, University of Geneva). The final construct was linearized using NcoI/SpeI and transfected into ΔKu80 parasites. Clones were obtained after mycophenolic acid selection and screened for locus insertion by PCR screen (see Figure S2C and S2D). Mutants were grown in 0.5 µg/ml of ATc (Sigma-Aldrich) for 1–4 d, total RNA was isolated (RNAeasy, Qiagen), reverse transcribed (Invitrogen), and reverse transcription-PCR was performed using SFA2 specific and control primers (Table S1).

For SFA3 a cosmid (PSBLE51) was modified by recombineering [26] to replace the native promoter by a regulatable promoter. A suitable cassette was constructed by inserting a gentamycin marker [26] into the promoter replacement plasmid *pDT7S4_087270* [24]. The resulting plasmid (*pGDT7S4_087270*) was used as template to amplify the modification cassette using 50-bp homology flanks for insertion into SFA3 (Figure S2A). The modified cosmid was isolated by double selection on gentamycin and kanamycin [26] and transfected into TATiΔKu80 parasites [24], clones were isolated after pyrimethamine selection and tested for promoter replacement by PCR (Figure S2A and S2B).

### Protein Expression and Antibody Production

The complete coding region of TgSFA3 was amplified from the *T. gondii* genomic DNA and inserted into plasmid pAVA-421 6xHis [59]. Recombinant fusion protein was purified on Ni^2−^-NTA resin (Qiagen) [60]. Rabbits were immunized with 1 mg of purified protein, and serum was collected after 10 wk (Cocalico Biologicals). The sequence encoding for amino-acids 1–110 of TgCENH3 [9] was amplified and cloned into the same expression vector and purified in a similar fashion as SFA3. Mice were immunized with 0.4 mg of purified protein, and serum was collected after 10 wk.

### Fluorescence Microscopy

For IFAs, host cells (HFF) were inoculated onto coverslips and infected with parasites. Coverslips were fixed 24 h after infection with 4% formaldehyde in PBS and permeabilized with 0.2% Triton X-100 in PBS/3% BSA. Coverslips were then blocked in 3% bovine serum albumin (BSA) in PBS as previously described [61]. Primary antibodies used were mouse anti-alpha tubulin at a dilution of 1∶1,000 (12G10, a gift of Jacek Gaertig, University of Georgia), rabbit anti-Centrin1 at 1∶1,000 (gift of Iain Cheeseman, Massachusetts Institute of Technology), mouse anti-GFP at 1∶1,000–1∶400 (Torry Pines Biolabs), rat anti-HA at 1∶1,000 (clone 3F10, Roche Applied Science), mouse anti-IMC1 mAb 45.15 [62] at 1∶1,000 (gift of Gary Ward, University of Vermont), rabbit anti-IMC3 [63] at 1∶500, mouse anti-ISP1 mAb 7E8 [27] at 1∶1,000 (gift of Peter Bradley, University of California, Los Angeles), rabbit anti-MORN1 [30] at 1∶250, anti-aceytlated tubulin (Sigma) at 1∶1,000, and rabbit anti-SFA3 at 1∶1000 (generated in this study). The secondary antibodies used were AlexaFluor 350, AlexaFluor 488, and AlexaFluor 546 (Invitrogen), at a dilution of 1∶2,000. Images were collected on an Applied Precision Delta Vision inverted epifluorescence microscope using a UPlans APO 100×/1.40 oil lens. Time-lapse imaging was performed on the same Delta Vision microscope in a climate controlled chamber at 37°C. Cells were grown and imaged on glass bottom Wilco culture dishes (Wilco Wells). Images were obtained every 10 min for 4 h, and processed to correct for cell drifting. Photobleaching of SFA3-YFP was performed on a Delta Vision microscope using a single 600-ms pulse with a 488-nm laser set at 20% power, on a specified, diffraction-limited, region. Images were subjected to deconvolution and contrast adjustment using Applied Precision software (Softworx). For quantitative image analysis (presence/absence of fibers and number of nuclei/cell or centromeric clusters/nucleus, as described in the Results) a minimum of 50 vacuoles were scored for each out of at least three repeats. Averages and standard deviations were calculated and plotted using Graph Pad Prism Version 5.0c. Fiber length measurements were done using Volocity (Perkin-Elmer) on images taken of SFA3-YFP parasites under oryzalin or DMSO control treatment. Each point represents the average fiber length in one imaging field. Measurements were done for at least 18 fibers (and up to 81) per image. Four images from three independent replicates were used.

### Electron Microscopy

Cells infected with SFA2-HA parasites were fixed in 4% para-formaldehyde/0.05% glutaraldehyde in 0.1 M sodium phosphate buffer pH 7.4, blocked with 1% FBS in PBS (all RT), followed by overnight infiltration in 2.3 M sucrose/20% polyvinyl pyrrolidone at 4°C. Samples were frozen in liquid nitrogen, and sectioned with a Leica UCT cryo-ultra microtome. Sections were blocked with 1% FBS and subsequently incubated with rat anti-HA (1∶100), followed by incubation with rabbit anti-rat (1∶400), and finally 10 nM colloidal gold conjugated protein A. Washed sections were stained with 0.3% uranyl acetate/2% methyl cellulose and viewed with a JEOL 1200 EX transmission electron microscope. Controls, omitting the primary antibody, were consistently negative at the concentration of colloidal gold conjugated protein A used. Infected cells were also fixed in 2% glutaraldehyde in sodium phosphate buffer 0.1 M, pH7.4, followed by post-fixation with 1% osmium tetroxide in sodium phosphate buffer, alcohol dehydration, and Epon resin embedding. Serial sections were obtained with a Leica UCT cryo-ultramicrotome and collected in carbon-coated single hole grids.

### Western Blotting

Western blotting was performed as previously described [57]. We used anti-HA antibodies at a dilution of 1∶1,000, anti-tubulin at 1∶1,000, anti-GFP at 1∶500, and anti-SFA3 antibodies at a dilution of 1∶1,000. Pre-immune sera for anti-SFA3 antibodies were used at a dilution of 1∶1,000. Horseradish peroxidase (HRP)-conjugated anti-rat or anti-rabbit antibody (Pierce) was used at a dilution of 1∶20,000.

## Supporting Information

Figure S1 **Confirmation of the insertion of a triple hemagglutinin tag at the endogenous locus of TgSFA2.** (A) Southern blot of the parental strain, the polyclonal transfected population, and the SFA2-HA clone used in this study is shown. (B) A radioactively labeled probe complementary to the 3′ end of TgSFA2 was used for hybridization and is represented with a black bar. Insertion of the triple HA tag in the native locus creates an XhoI restriction site, absent in the parental cell line. This generates a 1.0-kb hybridization fragment in the SFA2-HA strain, while the probe hybridizes to a 4-kb fragment in the parental cell line. Note that in the polyclonal population both the 1.0- and 4.0-kb hybridization fragments are detected representing successful and unsuccessful insertions of the triple HA tag in the native locus, respectively. Also note additional integration products that were not further investigated.
(TIF)

Figure S2 **Schematic representation of the constructs engineered for obtain the iΔSFA3 and iΔSFA2 conditional knock out strains and PCR screens.** (A) Cosmid PSBLE51 containing the entire TgSFA3 gene (TgME49_ 018880) and several kilo bases upstream of the start codon was modified by recombineering a mutagenic cassette positioning an inducible promoter immediately upstream of the start codon, as well as a pyrimethamine resistance cassette for selection of transgenic parasites. Double homologous recombination of the construct into the TgSFA3 gene in the parasite yields iΔSFA3. The approximate location of primers used for PCR screen of clones is represented with arrow heads. (B) PCR screen of a successful transfectant shows that the TgSFA3 native locus is modified by insertion of a regulatable promoter between the endogenous promoter and the TgSFA3 gene coding sequence. The parental strain used for transfection (ΔKu80TaTi) is shown as a control. Note that primer pair 4/5 control for the absence of an episomal copy of the modified cosmid piΔSFA3 (double homologous recombination into the genome causes loss of the kanamycin cassette). (C) Schematic of the construct engineered to generate a conditional knockout of TgSFA2 (TgME49_005670), iΔSFA2. Upon successful insertion into the TgSFA2 locus, the expression of TgSFA2 is controlled by the inducible promoter T7S1, while the expression of the transactivating protein (TATi) is under the control of the TgSFA2 endogenous promoter. The approximate location of screening primers in the genome of the resulting mutant is represented with arrow heads. (D) PCR confirmation of successful insertion of the knock out construct piΔSFA2 into the TgSFA2 genomic context in the clone used for all experiments shown in this study. The parental strain used for transfection (ΔKu80) is shown as a control.
(TIF)

Table S1 **Name and sequence of all primers used in this study.**
(PDF)

Video S1 **SFAs form a dynamic structure and their expression is temporarily regulated.** Compilation of time-lapse images of SFA3-YFP parasites. Two parasite vacuoles are shown. The uppermost vacuole contains four parasites, while the lowermost vacuole contains two. Parasites outlines were obtained from overlaying fluorescence images with DIC images prior to compilation of the movie. Images were taken every 10 min for 2.5 h. At the beginning, SFA3-YFP signal can be detected in the uppermost vacuole as two dots per parasite. With time, these dots evolve into more elongated structures. At a later time point, the SFA3-YFP signal starts appearing in the lowermost vacuole. As the signal dissipated in the uppermost vacuole (presumably upon completion of division), the dots in the lowermost vacuole evolve into more elongated fiber like structures, which ultimately disappear as well. Note that not all parasites remain in the focal point for every time point imaged.
(MOV)

Video S2 **The SFA fiber is dynamic and grows off the centrosome in dividing parasites.** Compilation of time-lapse images of a single SFA3-YFP (green) parasites transiently co-expressing Centrin1-RFP (red). Centrin1 is a marker for the centrosome. Note that only one fiber and one centrosome appear in the focal plane. Images were taken every 10 min for 4 h. Rapid elongation of the SFA fiber, away from the centrosome, can be seen. The structure elongates up to 1 µm, at which point it appears to break. Following breakage, two structures can be seen. One is putatively associated with the tip of the daughter cell, while most of the structure remains associated to the centrosome. The structure disappears after rapid depolymerization following the breakage.
(MOV)

## Abbreviations

ATc: anhydrotetracycline
CenH3: centromeric variant histone H3
FNR-RFP: ferredoxin/NADPH reductase-RFP
HA: hemagglutinin epitope
IFA: immunofluorescence assay
IMC: inner membrane complex
ISP1: IMC subcompartment protein 1
MTOC: microtubule organizing center
RFP: red fluorescent protein
SFA: striated fiber assemblin
YFP: yellow fluorescent protein
