# Supplementary figures and images for: Cell Division in Apicomplexan Parasites Is Organized by a Homolog of the Striated Rootlet Fiber of Algal Flagella
Source: PLoS Biol. 2012 Dec 11;10(12):e1001444. doi: 10.1371/journal.pbio.1001444 (PMC3519896; doi:10.1371/journal.pbio.1001444)

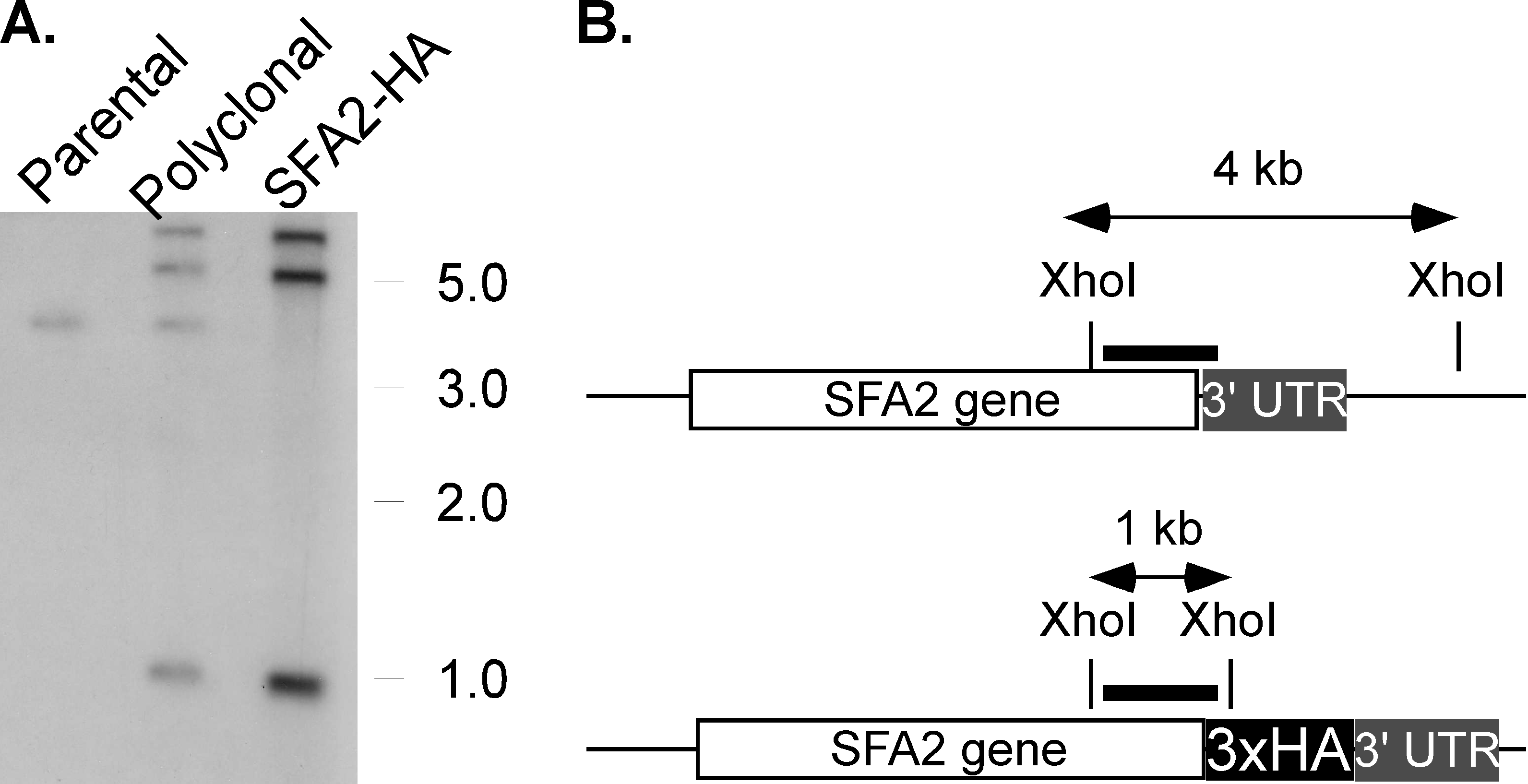

Supplement: Figure S1 — Confirmation of the insertion of a triple hemagglutinin tag at the endogenous locus of TgSFA2. (A) Southern blot of the parental strain, the polyclonal transfected population, and the SFA2-HA clone used in this study is shown. (B) A radioactively labeled probe complementary to the 3′ end of TgSFA2 was used for hybridization and is represented with a black bar. Insertion of the triple HA tag in the native locus creates an XhoI restriction site, absent in the parental cell line. This generates a 1.0-kb hybridization fragment in the SFA2-HA strain, while the probe hybridizes to a 4-kb fragment in the parental cell line. Note that in the polyclonal population both the 1.0- and 4.0-kb hybridization fragments are detected representing successful and unsuccessful insertions of the triple HA tag in the native locus, respectively. Also note additional integration products that were not further investigated. (TIF) [file pbio.1001444.s001.tif]

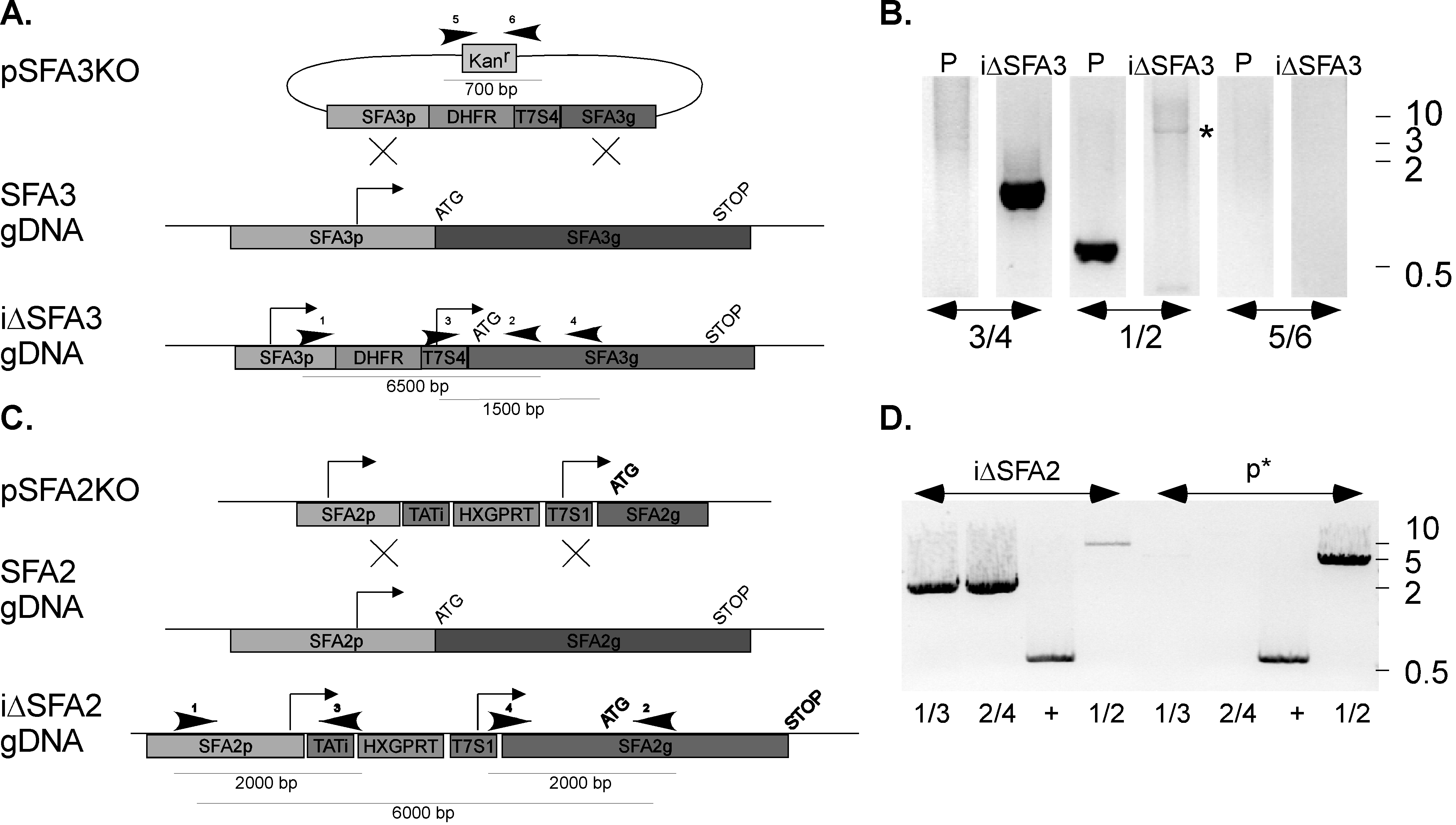

Supplement: Figure S2 — Schematic representation of the constructs engineered for obtain the iΔSFA3 and iΔSFA2 conditional knock out strains and PCR screens. (A) Cosmid PSBLE51 containing the entire TgSFA3 gene (TgME49_ 018880) and several kilo bases upstream of the start codon was modified by recombineering a mutagenic cassette positioning an inducible promoter immediately upstream of the start codon, as well as a pyrimethamine resistance cassette for selection of transgenic parasites. Double homologous recombination of the construct into the TgSFA3 gene in the parasite yields iΔSFA3. The approximate location of primers used for PCR screen of clones is represented with arrow heads. (B) PCR screen of a successful transfectant shows that the TgSFA3 native locus is modified by insertion of a regulatable promoter between the endogenous promoter and the TgSFA3 gene coding sequence. The parental strain used for transfection (ΔKu80TaTi) is shown as a control. Note that primer pair 4/5 control for the absence of an episomal copy of the modified cosmid piΔSFA3 (double homologous recombination into the genome causes loss of the kanamycin cassette). (C) Schematic of the construct engineered to generate a conditional knockout of TgSFA2 (TgME49_005670), iΔSFA2. Upon successful insertion into the TgSFA2 locus, the expression of TgSFA2 is controlled by the inducible promoter T7S1, while the expression of the transactivating protein (TATi) is under the control of the TgSFA2 endogenous promoter. The approximate location of screening primers in the genome of the resulting mutant is represented with arrow heads. (D) PCR confirmation of successful insertion of the knock out construct piΔSFA2 into the TgSFA2 genomic context in the clone used for all experiments shown in this study. The parental strain used for transfection (ΔKu80) is shown as a control. (TIF) [file pbio.1001444.s002.tif]
