# Supplementary material for: Cell Division in Apicomplexan Parasites Is Organized by a Homolog of the Striated Rootlet Fiber of Algal Flagella
Source: PLoS Biol. 2012 Dec 11;10(12):e1001444. doi: 10.1371/journal.pbio.1001444 (PMC3519896; doi:10.1371/journal.pbio.1001444)
Supplement: Table S1 — Name and sequence of all primers used in this study. (PDF) [file pbio.1001444.s003.pdf]

Number	Primer Name	Primer Sequence 5'-3'
1	SFA2 LIC F	tacttccaatccactatggggcaagg
2	SFA2 LIC R	tcctccagttccaa tttcgaaggccc
3	SFA3 LIC F	tacttccaatccaatttaatgcggccttgcttggacaatgtacc
4	SFA3 LIC R	tcctccacttccaattttagcggctgtcgtgacgagacg
5	SFA2 3' F	gctaaatcatctgaaaaaggacatcg
6	SFA2 3' R	cagcgagtgcttatatctgtggaac
7	SFA2 Prom F	agatctatggcgggggctgcagggtcgtgca
8	SFA2 Prom R	actagtctcgagctcaaactgttccttcagcg
9	SFA2 Gene F	ccatggcacgtgtaaaccttgtcgaagcag
10	SFA2 Gene R	gctagcaatgcacggccctcctgtggaaa
11	SFA2KO Screen 1	catatgcacacatataccaagacaccgggaa
12	SFA2KO Screen 2	cctaggtggagcctgccaaaaccgctcacat
13	SFA2KO Screen 3	tgagcgagtttccttgtcgtcag
14	SFA2KO Screen 4	atggagcagaagctcatctccgag
15	SFA2 RT PCR F	cgctgaaggaacagtttgagctcga
16	SFA2 RT PCR R	ggctgtcgtgtactggttgatagca
17	RT PCR Control F	tcgatacatttcgttcgcgtag
18	RT PCR Control R	tcgtccgctctgtcgccttgc
19	Gent F	gggattaatgcggccggccgctgaagttcc
20	Gent R	cccattaattgcaggaagttcctattctctagaaag
21	SFA3 Cosmid F	cagtccacgcggtcgaagctcgcggtgttgagcgaacggattcatggtttgaatggtaaccgacaaacgcgttc
22	SFA3 Cosmid R	gctttcgtctgtcttcaaccagatcttgaaaagcagatggagtctgaggccaagcagagaagggagagtgaagag a
23	SFA3KO Screen 1	atcaaggaagccatcacga

24	SFA3KO Screen 2	cacgaagatggaagacagc
25	SFA3KO Screen 3	cgccttggcgaatgttcatgac
26	SFA3KO Screen 4	gtgttcacgggtcgaagggataaa
27	SFA3KO Screen 5	atgattgaacaagatgg
28	SFA3KO Screen 6	tcagaagaactcgtc
29	SFA3 pAVA F	gggtcctggttcgctgaggccaagca
30	SFA3 pAVA R	cttgttcgtgctgtgtcgtgacgaga
